# Supplementary figures and images for: A hyperinflammation clinical risk tool, HI5-NEWS2, stratifies hospitalised COVID-19 patients to associate risk of death and effect of early dexamethasone in an observational cohort
Source: PLoS One. 2023 Jan 17;18(1):e0280079. doi: 10.1371/journal.pone.0280079 (PMC9844906; doi:10.1371/journal.pone.0280079)

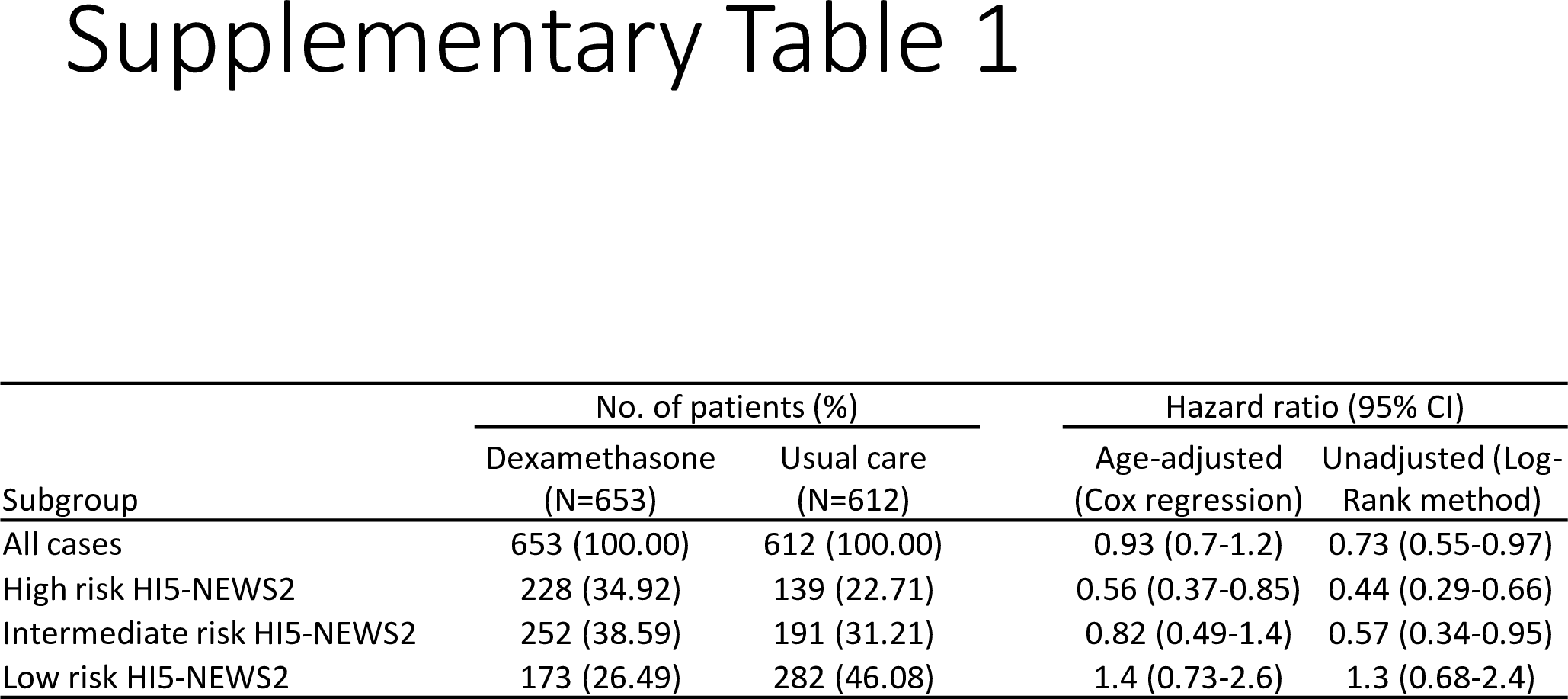

Supplement: S1 Table — Unadjusted hazard rates (log-rank method) for all cases, High HI5, High NEWS2, and HI5-NEWS2 High risk, Intermediate risk and Low risk cases, versus age adjusted analysis (Cox regression). (TIF) [file pone.0280079.s001.tif]

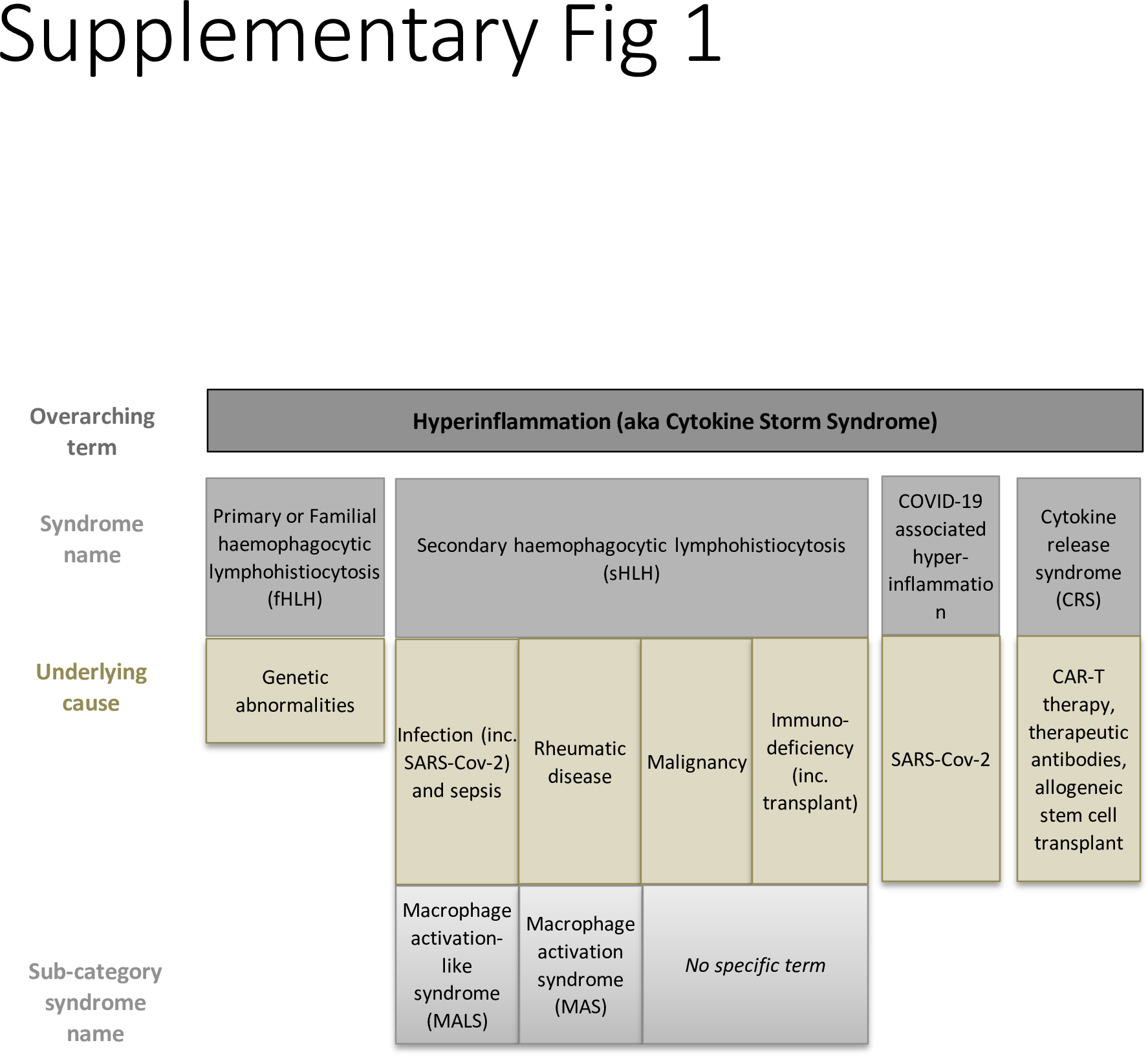

Supplement: S1 Fig — Hyperinflammation is induced as a consequence of a variety of underlying causes including SARS-CoV-2 [46]. (TIF) [file pone.0280079.s002.tif]

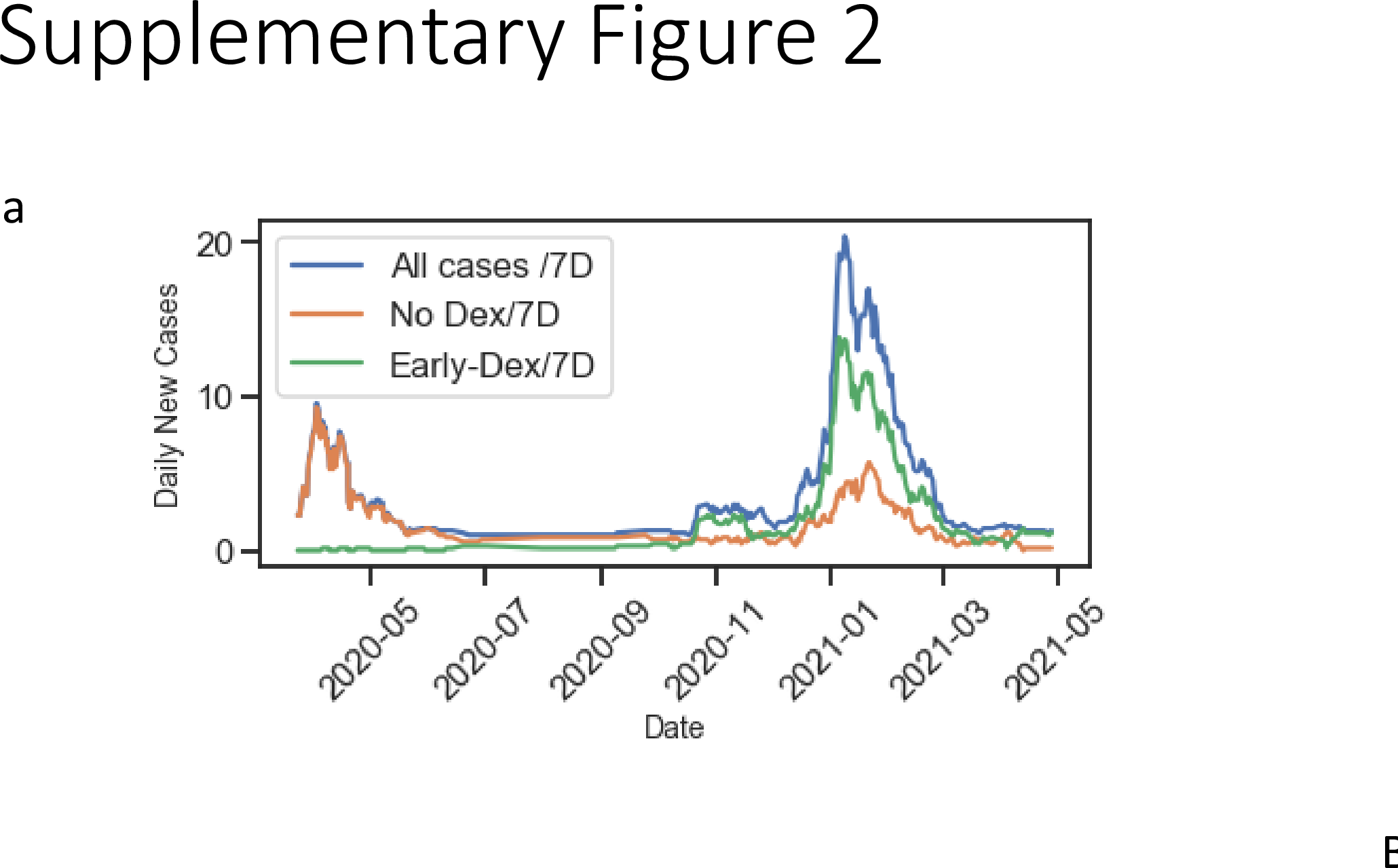

Supplement: S2 Fig — The admission of COVID-19 cases by laboratory confirmed (Sars-CoV-2) polymerase chain reaction confirmation date (X-axis), vs 7 day rolling average of number of cases (Y-axis). Blue line, all cases admitted to the institution; Orange line, cases not treated with dexamethasone recruited in this study; green line, cases receiving prescription for dexamethasone within day -1 to 2 of virus confirmation. (TIF) [file pone.0280079.s003.tif]

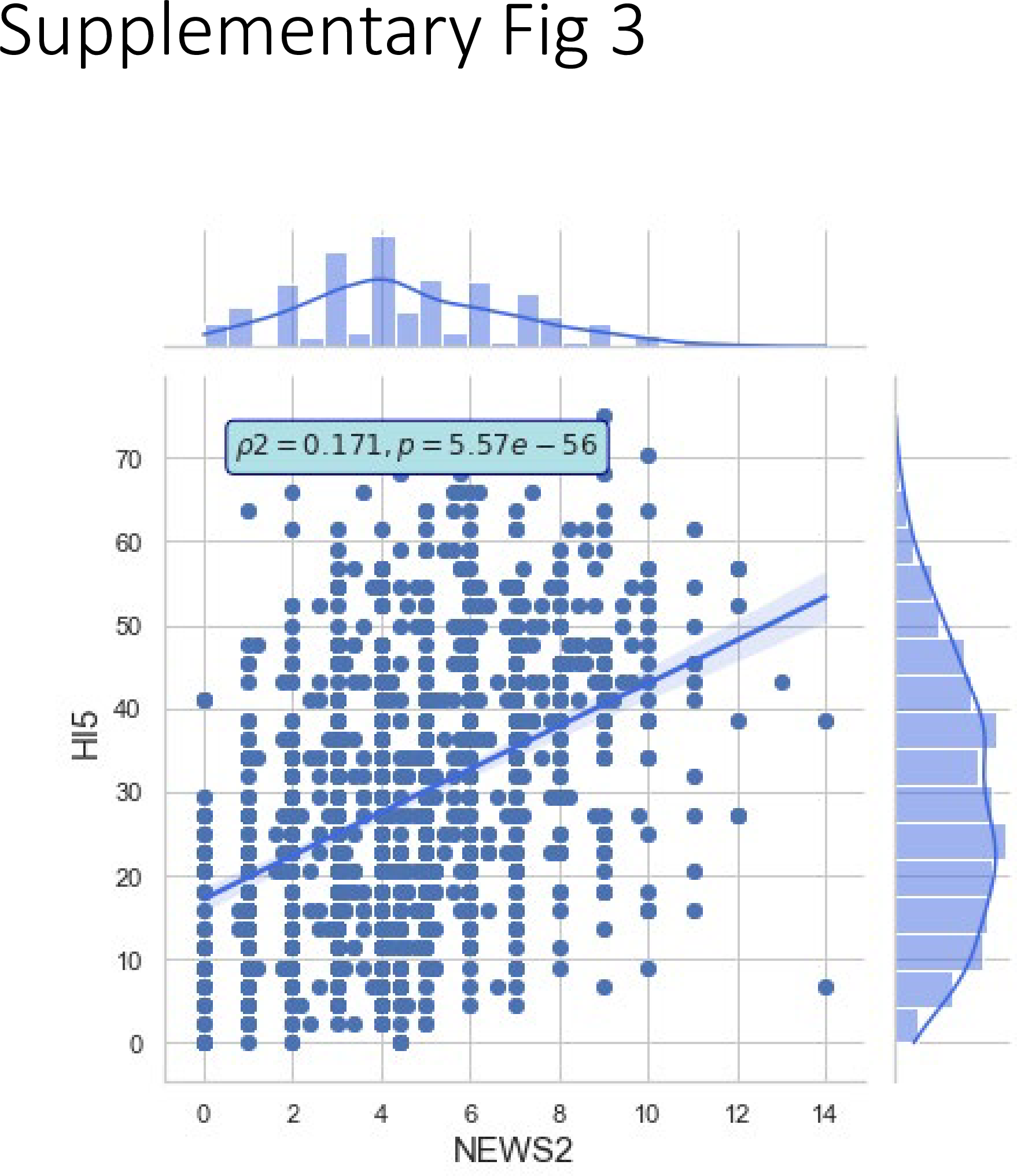

Supplement: S3 Fig — Correlation between HI5 (x-axis) and NEWS2 (y-axis). Pearson’s correlation ρ2 reported in the figure. (TIF) [file pone.0280079.s004.tif]

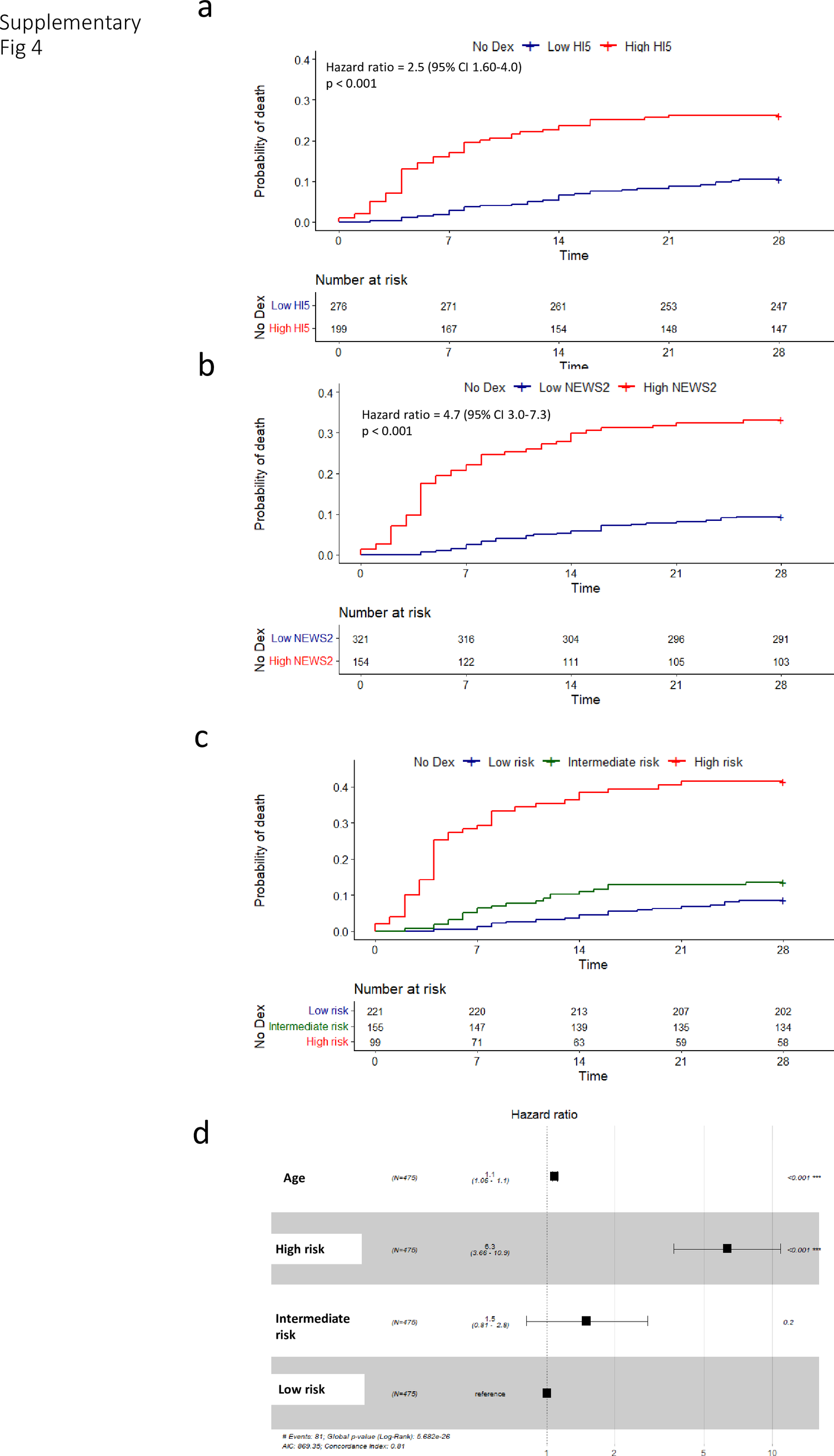

Supplement: S4 Fig — Kaplan–Meier survival curves for 28-day mortality among patients who were not treated with dexamethasone with high (red line) or low (blue line) HI5 scores (a) or NEWS2 scores (b). Panel C, Kaplan-Meier survival curves for cases classified as high risk (High HI5 and High NEWS2, red line), intermediate risk (High HI5 or High NEWS2, green line), and low risk (Low HI5 and Low NEWS2, blue line). (d) Cox regression analysis for Age, and HI5-NEWS2 risk status. All quoted hazard ratios are adjusted for age. At risk data are listed beneath plots. Time measured in days. Cases censored before 28 days indicated by +. (TIF) [file pone.0280079.s005.tif]

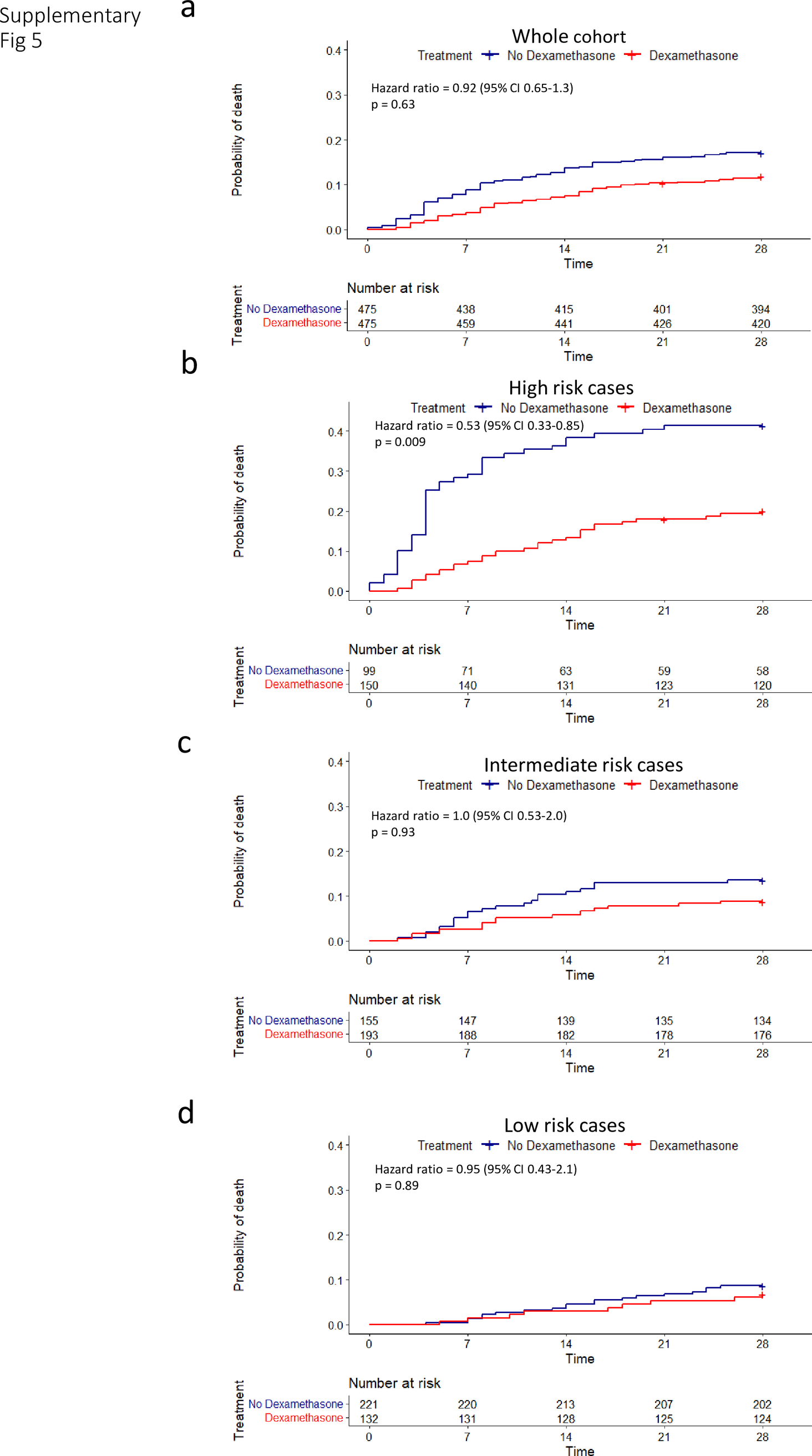

Supplement: S5 Fig — (a) Kaplan–Meier survival curves for 28-day mortality among the whole cohort in those who were treated with dexamethasone (red line) vs untreated cases (blue line). (b-d) Kaplan–Meier survival curves for those treated with dexamethasone (red lines) vs untreated (blue lines) in high risk (b), intermediate risk (c) or low risk (d) groups. All quoted hazard ratios are adjusted for age. At risk data are listed beneath plots. Time measured in days. (TIF) [file pone.0280079.s006.tif]
